# Integrative genomic and functional analysis of human oral squamous cell carcinoma cell lines reveals synergistic effects of *FAT1* and *CASP8* inactivation

**DOI:** 10.1016/j.canlet.2016.09.014

**Published:** 2016-12-01

**Authors:** Tyler F. Hayes, Nathan Benaich, Stephen J. Goldie, Kalle Sipilä, Ashley Ames-Draycott, Wenjun Cai, Guangliang Yin, Fiona M. Watt

**Affiliations:** a Centre for Stem Cells and Regenerative Medicine, King's College London, 28th Floor, Tower Wing, Guy's Hospital, London SE1 9RT, UK; b Department of Medicine, Central Clinical School, Level 6, Alfred Centre, 99 Commercial Road, Melbourne, VIC 3004, Australia; c BGI-Shenzhen, Shenzhen 518038, China

**Keywords:** Oral squamous cell carcinoma, Caspase 8, FAT1, Whole exome sequencing

## Abstract

Oral squamous cell carcinoma (OSCC) is genetically highly heterogeneous, which contributes to the challenges of treatment. To create an *in vitro* model that accurately reflects this heterogeneity, we generated a panel of HPV-negative OSCC cell lines. By whole exome sequencing of the lines and matched patient blood samples, we demonstrate that the mutational spectrum of the lines is representative of primary OSCC in The Cancer Genome Atlas. We show that loss of function mutations in *FAT1* (an atypical cadherin) and *CASP8* (Caspase 8) frequently occur in the same tumour. OSCC cells with inactivating *FAT1* mutations exhibited reduced intercellular adhesion. Knockdown of *FAT1* and *CASP8* individually or in combination in OSCC cells led to increased cell migration and clonal growth, resistance to Staurosporine-induced apoptosis and, in some cases, increased terminal differentiation. The OSCC lines thus represent a valuable resource for elucidating the impact of different mutations on tumour behaviour.

## Highlights

• Generation of human oral squamous cell carcinoma lines with whole exome sequence data.
• Mutational spectrum of the lines is representative of primary tumours.
• Loss of function mutations in FAT1 and CASP8 frequently co-occur.
• Inactivating FAT1 mutations correlate with reduced intercellular adhesion.
• Inactivation of FAT1 and CASP8 results in increased clonal growth, migration and apoptosis resistance.

## Introduction

The global annual incidence of head and neck squamous cell carcinoma (HNSCC) is approximately 600,000, with over 300,000 deaths attributable to the disease each year [1]. HNSCC lesions frequently recur and metastasize to other locations, contributing to significant morbidity and mortality [2]. Despite improvements in surgical care and adjuvant therapy, the 5-year survival rate is only 60% in the United States [3].

Previous studies have used next-generation whole exome sequencing of primary tumours to reveal the mutational landscapes of human HNSCCs and suggest novel oncogenic drivers to target for potential therapies [4], [5], [6]. HNSCC is strikingly heterogeneous at both the genetic and the cellular level. No single genetic lesion predominates and patients often develop more than one primary tumour.

Oral cancer is a subtype of HNSCC, comprising tumours of the lip and oral cavity. Worldwide, over 250,000 new cases and 125,000 deaths from oral cancer occurred in 2008 [1]. Approximately 90% of oral cancers are squamous cell carcinomas (SCCs), arising in the multilayered epithelia that line the mouth and lips. Oral SCC (OSCC) frequently involves the tongue, and can also occur in the floor of the mouth, gingiva, cheek and palate. The two major risk factors for OSCC are smoking and alcohol consumption [7]. In contrast to HNSCC of the tonsils and oropharynx, OSCC tends not to be associated with HPV infection [8], [9]. Despite advances in treatment, the survival rate for OSCC remains stubbornly low [3].

As in other solid tumours [10], [11], the properties of the malignant epithelial cells in HNSCC elicit an immune infiltrate and activation of fibroblasts in the surrounding connective tissue stroma [1], [3], [12]. Integrative analysis of the genetic and cellular heterogeneity of HNSCC is beginning to reveal new biologically distinct subtypes of the disease [13], and greater understanding of their significance could inform future therapies. One way to achieve this is by studying the properties of cells isolated from HNSCC.

Cells can readily be cultured from human oral tumours and normal oral mucosa, and several well-annotated collections of HNSCC cells are available [14], [15], [16]. However, genetic changes in individual cell lines have not been compared with control DNA from the original donors. Thus it has been difficult to discover whether the lines truly reflect the genetic heterogeneity of OSCC or are dominated by mutations acquired during long-term culture. The value of such lines lies in characterising the biology of genes that are frequently mutated in OSCC, including *NOTCH1*, *CASP8* (Caspase 8) and *FAT1* (an atypical cadherin) [6], which are not conventional oncogenes/tumour suppressor genes and can potentially have pleiotropic effects on tumour properties. For example, the association between *FAT1* mutation and overall survival in HNSCC differs according to the HPV status of the tumour [17], and loss of Caspase 8 not only has cell intrinsic effects [18] but can also trigger inflammation [19]. Furthermore, there is evidence for biological interactions between FAT1 and Caspase 8, with FAT1 acting as an antagonist of Caspase 8 in a synthetic lethal screen in cancer cell lines [20].

In this study, we set out to develop new OSCC lines, discover which mutations are tumour-acquired and determine whether they are indeed representative of mutational burden in primary tumours. We then used the lines to explore the impact of mutations in *CASP8* and *FAT1* on cell behaviour.

## Materials and methods

### Derivation of OSCC lines

Anonymized biopsies of OSCC or normal oral mucosa were collected with appropriate ethical approval (UK National Research Ethics Service (08/H0306/30). Cells were isolated and cultured on a feeder layer of J2 3T3 cells in complete FAD medium as described previously [16].

### Whole exome sequencing

Genomic DNA was extracted from OSCC lines (passage 2–4) and patient-matched blood. Whole exome sequencing was performed by Beijing Genomics Institute (Hong Kong). Raw image files were processed by Illumina base calling Software 1.7 or base calling with default parameters, and the sequences of each individual were generated as 90 bp paired-end reads. High-quality reads were aligned against the NCBI human reference genome (hg19) using Burrows-Wheeler Aligner (v0.5.9) with default parameters. Picard (v1.54) was employed to mark duplicates and was followed by Genome Analysis Toolkit (v1.0.6076, GATK IndelRealigner) to improve alignment accuracy. Putative somatic single nucleotide variations (SNVs) were predicted by VarScan2.25 with the parameters as “-- min-coverage 5 --min-coverage-normal 5 --min-coverage-tumour 5 --min-var-freq 0.1 --min-freq-for-hom 0.75 --min-avg-qual 0 —somatic-p-value 0.15”. In order to obtain high confidence somatic SNVs, an in-house pipeline was applied. Somatic InDels were predicted by GATK SomaticInDelDetector with default parameters. A pipeline was developed to obtain high confidence somatic InDels; normal and tumour bam were reused to perform local realignment and germline indels were filtered for high confidence indels, with normal coverage and tumour coverage no less than 5. High confidence somatic single nucleotide variants and InDels were annotated using ANNOVAR. Functional impacts of missense mutations were predicted using SIFT, PolyPhen2, PhyloP, MutationTaster and LRT annotations.

### Prediction of driver genes and pathways

The Oncodrive-fm method was applied, as previously published, to identify significantly mutant genes and Kyoto Encyclopedia of Genes and Genomes (KEGG) pathways [21]. Pathway enrichment analysis was also performed to identify additional significantly mutated KEGG pathways.

### KEGG pathway analysis and clustering

Whole exome sequencing data from The Cancer Gene Atlas (TCGA) HNSCC collection [6] were accessed from cBioPortal.org. KEGG pathway analysis was performed; –log_2_(*P*) values were calculated and presented in heat maps created using GENE-E software (Broad Institute). OncoPrint, mutation diagrams and tests for mutual exclusivity of genomic alterations were downloaded from cBioPortal.

### Quantitative real-time RT-PCR

RNA was harvested using the RNeasy Mini Kit and QIAshredder columns according to the manufacturer's instructions (Qiagen). cDNA was synthesized using SuperScript™ III First-Strand Synthesis SuperMix (Invitrogen). qPCR was performed using TaqMan Fast Universal PCR Master Mix without AmpErase UNG (Life Technologies) on the CFX96 Touch Real-Time PCR Detection System (Bio-Rad) with the following primers: CASP8 (Hs01018151_m1), FAT1 (Hs00170627_m1) and GAPDH (Hs02758991_g1) as the internal control (Life Technologies). Relative quantification was performed using the 1/ΔCt or ΔΔCt methods.

### Transient transfection

SMARTpool ON-TARGETplus CASP8 and FAT1 (GE Healthcare) and Silencer^®^ Negative Control No. 1 (Thermo Fisher) siRNAs were used. Prior to transfection, wells were coated with 20 μg/mL Rat Tail Collagen I (Corning) and incubated for 1 h at 37 °C. Cells were transfected using either Lipofectamine RNAiMax or Lipofectamine 2000 with Opti-MEM medium (Thermo Fisher). The viability of transfected cells was confirmed by Trypan Blue exclusion prior to performing cell behaviour assays.

### Colony formation

48 h after transfection, cells were seeded at clonal density in 6-well plates on a layer of feeders. 10–14 days after seeding, feeders were detached and keratinocytes were fixed in 10% formalin and stained with a solution of 1% Rhodamine B and 1% Nile blue. Well images were acquired on the Gel Doc™ XR+ (Bio-Rad). Percent well area covered by cells was determined using the ColonyArea plugin [22] for ImageJ.

### Cell migration assay

24 h after transfection, cells were seeded on collagen-coated well plates in the Oris™ Cell Migration Assay (Platypus Technologies) and cultured in complete FAD medium. 24 h after plating, cell seeding stoppers were removed. Phase-contrast images were acquired for 16–24 h using an IncuCyte^®^ ZOOM (Essen BioScience). Area closure was quantified in ImageJ, following the manufacturer's instructions.

### Induction of apoptosis

24 h after transfection, cells were seeded on collagen-coated 96-well plates in complete FAD medium. 24 h later cells were incubated with varying concentrations of Staurosporine (1285; Tocris) in complete FAD medium for 4 h at 37 °C.

### Immunofluorescence staining

Cells were fixed in 4% paraformaldehyde, permeabilized using 0.5% Triton X-100 (Sigma-Aldrich) in PBS and blocked in a solution of 10% fetal bovine serum (Thermo Fisher), 0.25% gelatin from cold water fish skin (Sigma-Aldrich) and 0.5% Triton X-100 in PBS. Cells were incubated overnight at 4 °C with the following primary antibodies in blocking buffer: rabbit anti-Ki67 (1:50; ab16667; Abcam), Alexa Fluor^®^ 488 conjugated anti-cleaved Caspase-3 (Asp175) antibody (1:50 in blocking buffer; 9669; Cell Signalling) or mouse anti-transglutaminase 1 (TGM1; clone BC1; 1:500). Cells were incubated for 1 h at room temperature in blocking buffer with the following secondary antibodies, as appropriate: Alexa Fluor^®^ 488 goat anti-rabbit IgG H + L (1:1000; ab150077; Abcam) or Alexa Fluor^®^ 555 goat anti-mouse IgG H + L (1:1000; ab150114; Abcam). Plasma membranes and nuclei were counterstained with CellMask™ Deep Red (1:1000 in PBS; C10046; Thermo Fisher) and DAPI (1 μg/mL in PBS), respectively, for 1 h at room temperature. Following Caspase-3 labelling cell nuclei were counterstained with DRAQ5™ (5 μM in PBS; 108410; Abcam). Images were acquired using the Operetta^®^ High-Content Imaging System (PerkinElmer). Positive nuclear Ki67 and cytoplasmic TGM1 immunostaining were quantified using an in-house pipeline on Harmony^®^ software (PerkinElmer), while Caspase-3 positive cells were quantified using the “Apoptosis-1” module (PerkinElmer).

### Statistical analyses

Data were analyzed using an unpaired Student's t-test or one-way ANOVA with Tukey's Multiple Comparison Post Test on GraphPad Prism 5.0b, and a *P* value of <0.05 was considered significant, unless otherwise noted.

## Results

### Whole exome sequencing of OSCC lines

**Fig. 1:**
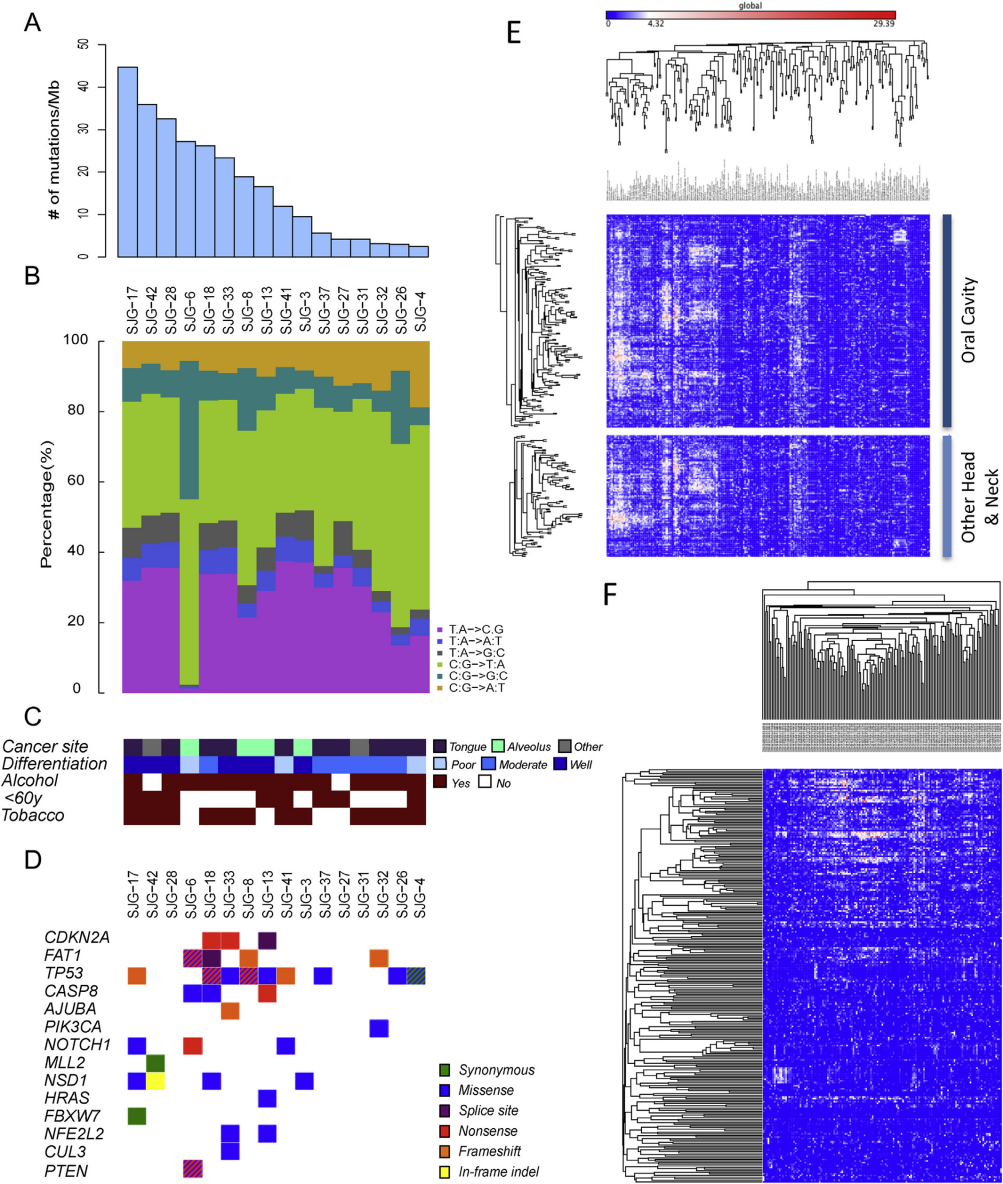
Genomic analysis of OSCC lines and TCGA HNSCC tumours. A–C) Somatic mutation rates (A), nucleotides transition and transversion frequencies (B), clinical characteristics and social histories (C) of cell lines and patients from which they were derived. D) Somatic single nucleotide variants (SNVs) and insertion/deletions (indels) in significantly mutated genes. Genes are ranked in order of significance from TCGA (6). E) Unsupervised clustering of TCGA HNSCC samples according to significance of mutated KEGG pathways. Rows are individual tumours; columns are KEGG pathways. OSCC are shown separately from other HNSCC. F) Unsupervised clustering of TCGA OSCC samples ranked according to frequency of mutated KEGG pathways (descending rank order) in OSCC cell lines. Rows are pathways and columns are individual tumours. (E, F) Colour scale is based on –log_2_(*P*) values.)

We derived multiple low passage polyclonal cell lines from primary oral squamous cell carcinoma biopsies by culture on a 3T3 J2 feeder layer in order to minimise selection for rapidly dividing cells [16]. Whole exome sequencing was performed on 16 lines, together with patient-matched blood. We achieved 37- and 43-fold mean sequence coverage of targeted exonic regions, with 73 and 77% of loci covered at ≥20-fold from tumour and matched blood samples, respectively (Supplementary Fig. S1). Mutation rates varied from 2.50 to 44.7 mutations/megabase (mean 16.9 ± 13.5), with 80–1431 somatic mutations per sample (mean 539 ± 432) (Fig. 1A; Supplementary Table S1). A total of 8629 single nucleotide variations across 2611 genes were found, of which 5839 (68%) were synonymous, 2621 (30%) nonsynonymous, 125 (1.4%) stop-gains and 42 (0.49%) were splice-site mutations (Supplementary Table S2). Ninety-five insertions/deletions (indels) were found across 83 genes, of which 36 (43%) and 27 (32.5%) were non-frameshift and frameshift deletions, respectively. Thirteen (16%) and 19 (23%) were non-frameshift and frameshift insertions, respectively. The ratio of nucleotide transitions to transversions ranged from 1.17 to 3.00 (mean 2.24 ± 0.436) (Fig. 1B). The frequency of C:G to A:T transversions and C:G to T:A transitions varied inversely with mutation rate, while the frequency of T:A to C:G transitions increased with mutation rate.

Ten of the 16 cell lines were derived from cancers of the tongue, four from the alveolus, one from the buccal mucosa and one from the hard palate (Fig. 1C; Supplementary Table S1). No associations were detected between self-described alcohol consumption, tobacco history or age, or between mutation rate or differentiation status of the primary tumour. However, well-differentiated tumours tended to have higher mutation rates than moderately differentiated tumours. All tumours were negative for common genotypes of human papilloma virus (HPV), determined by the absence of viral DNA integration in the sequenced exons.

To identify putative driver genes and pathways that, when mutated, contribute to OSCC tumourigenesis, the Oncogene-fm method was applied [21]. We considered significant only those mutated genes or Kyoto Encyclopedia of Genes and Genomes (KEGG) pathways with *Q* values < 0.10. Only two genes were found to be significantly mutated – the canonical tumour suppressor *TP53* (p-value 1.49 × 10^−9^; q-value 3.72 × 10^−7^) and *CSMD2* (CUB and sushi domain-containing protein 2; p-value 0.00024; q-value 0.03013). While *CSMD2* has a tumour suppressive role in several cancers [23], [24], *CSMD2* mutations in HNSCC have not been described previously.

Twenty KEGG pathways were significantly mutated across the 16 OSCC lines (Supplementary Table S3). Unsurprisingly, ten were pathways for specific cancers and three for other human diseases profiled in KEGG. Three cellular community pathways (Adherens Junction, Focal Adhesion and Tight Junction) and two signalling pathways (Wnt and B Cell Receptor Signalling Pathways) were also significantly mutated. Pathway enrichment analysis was performed in parallel, and seven KEGG pathways were significantly mutated with a corrected *P* value < 0.10 (Supplementary Table S4). Common pathways identified by both methods were Focal Adhesion and Wnt Signalling. The contribution of upregulated Wnt/β-catenin signalling to cell survival and invasion in HNSCC has been previously discussed [15], [25], [26]. Recent work suggests that transcriptional regulation of focal adhesion genes plays a crucial role in OSCC pathogenesis and that focal adhesion kinase (*FAK*) is a key mediator of extracellular matrix interactions and cellular signalling, contributing to HNSCC onset and progression [27], [28].

In 2015, The Cancer Gene Atlas (TCGA) Network published genomic profiles for 279 HNSCCs [6]. Of the 11 genes identified in that study as significantly mutated and eight trending towards significance, nine and five, respectively, were identified as harbouring somatic mutations across 13 of our 16 cell lines (Fig. 1D). These include inactivating *NOTCH1* mutations (found in 10–15% of HNSCC) [4], [5], [6], which cluster to the EGF-like binding domain and domains necessary for transactivation of target genes. Tumour-associated *NOTCH1* mutations were identified in three of our OSCC lines via whole exome sequencing (Fig. 1D). In addition, Sanger sequencing of the entire genomic region of *NOTCH1-3* was performed on all OSCC lines, including lines for which matched blood was not available. This identified several additional novel *bona fide* somatic (nonsynonymous, nonsense and indel) mutations (Supplementary Fig. 2). 44% of the cell lines contained mutations in *NOTCH1*, many of the mutations clustering in the EGF-like domain. Mutations in *NOTCH2* and *3* were less common. The presence of mutations in the PEST domain of *NOTCH3* could potentially result in increased receptor activity due to decreased ubiquitin-mediated degradation.

### OSCC lines are representative of primary tumour heterogeneity

To discover whether the mutational spectrum of OSCC is distinct from that of other HNSCCs, we determined the site of origin of each of 267 tumours in TCGA and then performed unsupervised clustering of the samples according to the significance of mutated KEGG pathways (Fig. 1E). This indicated that the spectrum of KEGG pathways mutated in OSCC was similar to that of HNSCC arising in other sites.

To determine whether the KEGG pathways that were mutated in the OSCC lines were similar to those in primary tumours, we performed unsupervised clustering of TCGA OSCC samples ranked according to the frequency of mutated KEGG pathways in the cell lines. As shown in Fig. 1F, the pathways that were mutated in the lines were indeed representative of those in primary tumours.

### Mutations in *CASP8* and *FAT1*

**Fig. 2:**
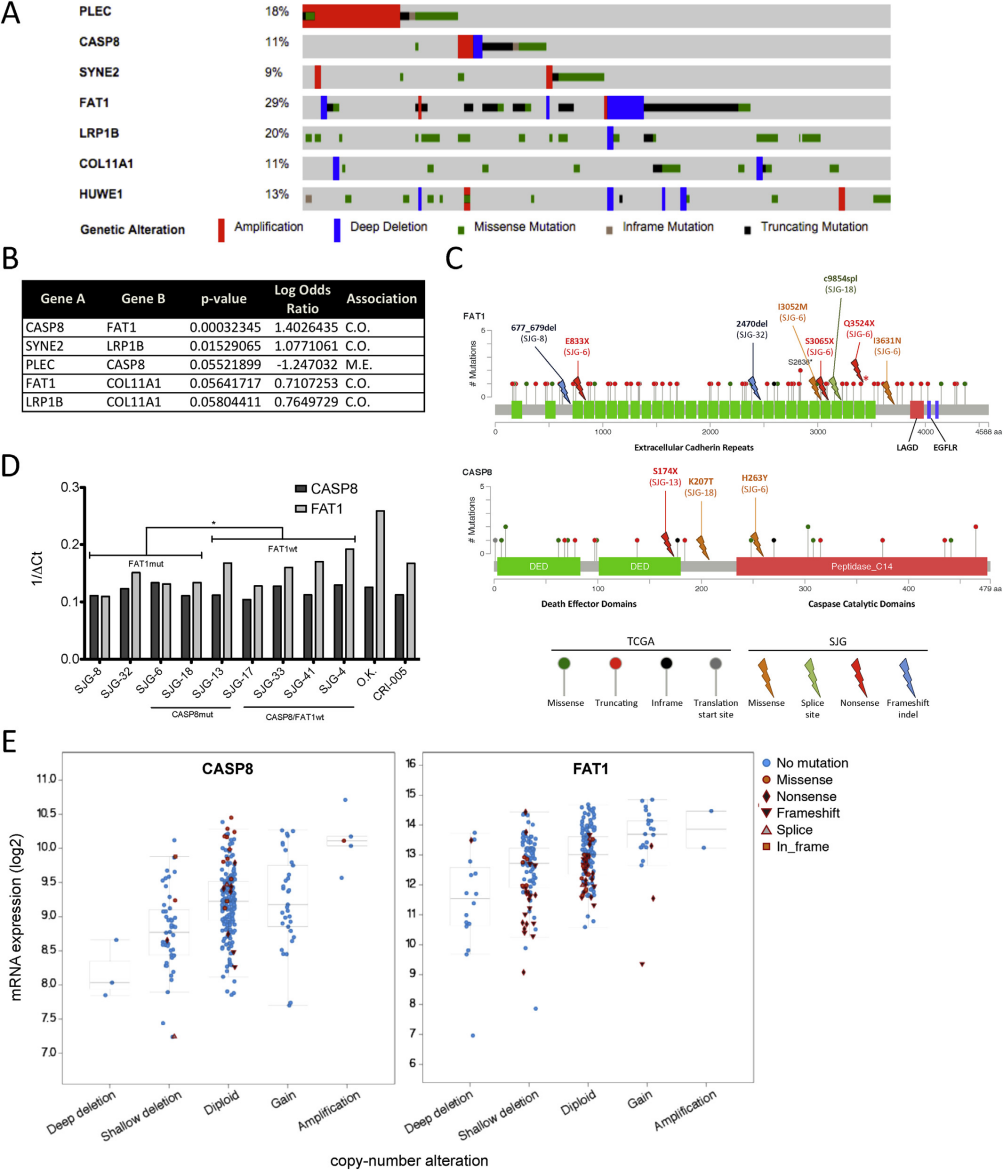
Identification of *CASP8* and *FAT1* as putative HNSCC/OSCC drivers. A) Genetic alterations in putative driver genes identified by exome sequencing and literature review. Samples are arranged to emphasize mutual exclusivity among mutations. Samples without genetic alterations in these genes are not shown. B) Pair associations of genetic alterations among putative driver genes in TCGA HNSCC project. *P* value was determined by Fisher's Exact test. C.O. = co-occurrence, M.E. = mutually exclusive. C) Genetic alterations in *CASP8* and *FAT1* in TCGA HNSCC and OSCC cell line datasets. LAGD = laminin a-g domain; EGFLR = EGF-like repeat; asterisk indicates mutation present in both TCGA and cell line datasets. D) mRNA expression of *CASP8* and *FAT1* in OSCC cell lines. *P* value was determined by unpaired Student's t-test. *P < 0.01–0.05. No significant difference was detected between presence of *CASP8* mutations and *CASP8* mRNA expression. OK and CRI-005 are two strains of primary oral keratinocytes. mut = mutant, wt = wild type. E) *CASP8* mRNA levels in tumours with mutant and wild type *CASP8* (left hand panel) and *FAT1* mRNA levels in tumours with mutant and wild type *FAT1* (right hand panel) stratified by putative copy-number alterations. Data from TCGA (6).

To evaluate the impact of mutations and expression of putative driver genes established by genomic studies on OSCC cell behaviour, we conducted a systematic literature review of genes implicated in homoeostasis or disease in stratified squamous epithelia through regulation of adhesion, invasion or motility. We focused on genes that were members of multiple mutated non-tissue specific KEGG pathways and/or were most significantly mutated in our study and TCGA. We prioritized those genes harbouring mutations in more than 8% of HNSCCs and at least two of our cell lines. Seven genes were identified – *PLEC*, *CASP8*, *SYNE2*, *FAT1*, *LRP1B*, *COL11A1* and *HUWE1* (Fig. 2A). We also queried TCGA for pair associations of mutations across these genes in HNSCC (Fig. 2B). *CASP8* and *FAT1* (*P* value = 0.0003), and *SYNE2* and *LRP1B* (*P* value = 0.015) were identified as frequently co-mutated in HNSCC.

Of the 279 patients with HNSCC reported by TCGA in 2015 [6], 172 patients had tumours of the oral cavity and the other 107 had tumours of the oropharynx (*n* = 33), hypopharynx (*n* = 2) and larynx (*n* = 72). Considering copy number alterations and truncating, missense and in-frame mutations, 23% of tumours of the oral cavity harboured mutations in *FAT1* alone, 5.8% in *CASP8* alone and 9.3% in both *FAT1* and *CASP8*, compared with 22%, 2.8% and 1.9%, respectively, in tumours of other areas of the head and neck. The increased frequency of *CASP8* and *FAT1* co-mutations in OSCC relative to other HNSCC, recent evidence suggesting functions of *CASP8* that extend beyond programmed cell death [29], and uncertainty surrounding the role of *FAT1* in oncogenesis [30] led us to focus on these two genes.

Whole exome sequencing revealed eight putative inactivating somatic mutations in *FAT1* across four of the 16 OSCC lines, and three in *CASP8* across three lines (Fig. 2C). Inactivating mutations were defined as frameshift indels, and splice-site, nonsense and missense mutations predicted to be deleterious to protein function by ANNOVAR. Two lines simultaneously harboured mutations in both *FAT1* and *CASP8*. Similar to the gene span of inactivating mutations in TCGA, mutations in *FAT1* and *CASP8* did not localize to any particular protein domain.

We next determined mRNA levels of *CASP8* and *FAT1* in OSCC cell lines by q-PCR (Fig. 2D). There was no significant difference between *CASP8* levels in wild type and mutant lines, nor between OSCC cells and normal oral keratinocytes. However, there was a trend for lower *FAT1* mRNA levels in *FAT1* mutant lines compared to wild type (*P* value < 0.05). In addition, *FAT1* mRNA levels were higher in normal oral keratinocytes than OSCC lines (Fig. 2D). These observations were consistent with primary tumour data from TCGA, since *CASP8* mRNA levels in tumours with mutant *CASP8* did not differ from those with wild type *CASP8* at a given copy number, whereas there was a trend to lower *FAT1* mRNA levels in tumours bearing *FAT1* mutations (Fig. 2E).

### Phenotypic effects of loss or inactivation of *CASP8* and *FAT1*

**Fig. 3:**
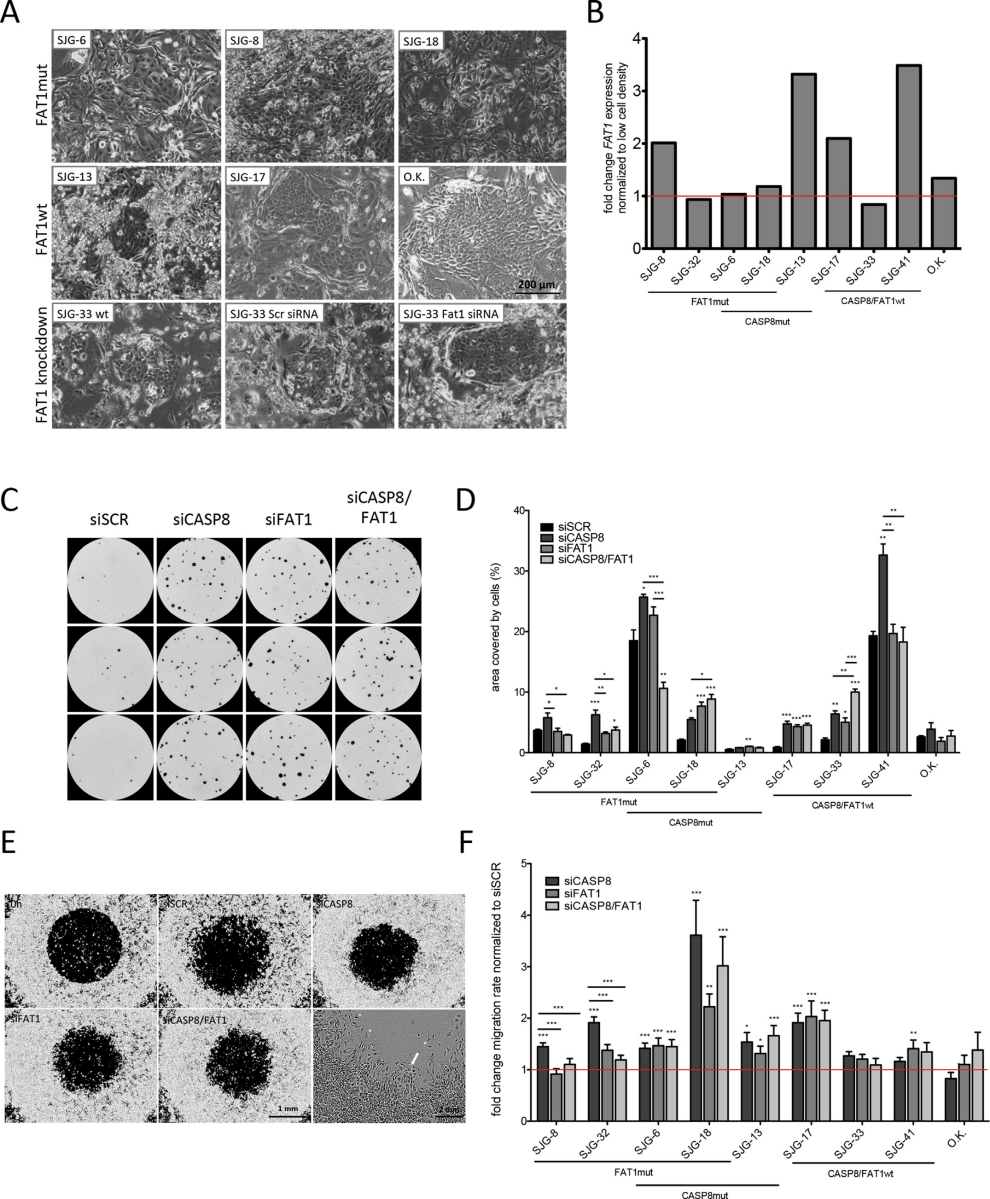
Phenotypic impact of *CASP8* and *FAT1* mutations and siRNA knockdown in OSCC lines. A) Phase-contrast microscopy of OSCC lines with (mt) and without (wt) predicted inactivating mutations in *FAT1*, and effects of *FAT1* siRNA on SJG-33 (wt for *FAT1*). B) *FAT1* mRNA expression in OSCC lines plated at low and high density for 48–72 h. OK = primary oral keratinocytes. C, D) Impact of *CASP8* and *FAT1* knockdown on clonal growth. (C) Triplicate dishes of SJG-17 cells. (D) Percent well area covered by cell clones. Significant differences from non-targeting control siRNA (siSCR) are shown. A–D) Cells were cultured on feeders. E, F) Impact of *CASP8* and *FAT1* knockdown on cell migration. (E) Representative images of cells at start (0 h) or 24 h later. In high magnification view, arrow indicates migratory cell front; asterisks indicate individual migrating cells. (F) Migration rate, defined as fold change in area of central detection zone at 16–24 h. Data are presented as mean ± SEM. (E, F) Cells were plated in the absence of feeders. (D, F) *P* values were determined by ANOVA and Tukey's test. *P < 0.01–0.05, **P < 0.001–0.01, ***P < 0.0001–0.001.

To characterise the effect of *FAT1* mutations on OSCC lines we first observed the morphology of cells grown on feeders. As shown in Fig. 3A, cells with *FAT1* mutations tended to form less cohesive colonies than cells that were wild type for *FAT1*. However, *FAT1* knockdown in SJG-33 cells, which are wild type for *FAT1*, was not sufficient to reduce cell–cell adhesion (Fig. 3A and B). We also observed that the level of *FAT1* mRNA increased with increased cell density in cells with wild type but not mutant *FAT1* (Fig. 3B). This did not correlate with the level of *FAT1* mRNA in cells at low density and was more marked in OSCC lines than in control oral keratinocytes.

To determine the impact of *FAT1* expression on the growth of OSCC cells in culture, we performed siRNA-mediated knockdown in cells with wild type FAT1 (Supplementary Fig. 3A) and then compared clonal growth with cells expressing scrambled control siRNA (Fig. 3C and D; Supplementary Fig. 3B). We also examined cells in which we knocked down *CASP8*, alone or in combination with *FAT1* knockdown. We observed that knockdown of each gene, alone or in combination, stimulated clonal growth of OSCC in culture but had no effect on the growth of primary oral keratinocytes.

Given the association between *FAT1* mutation and cell–cell adhesion (Fig. 3A) and between *CASP8* and integrin-mediated cell migration [29], we next examined the effect of knockdown on cell migration. We monitored the ability of cells to migrate into a circular area exposed in the centre of the culture dish during a 24 h period. In the case of mutant cells, there were frequent individual cells that had migrated into the area independently of the migrating epithelial front (Fig. 3E). Quantitation of the assays showed that knockdown of *FAT1* or *CASP8* alone and in combination increased cell migration in OSCC but not in primary keratinocytes (Fig. 3F). In some cases the effect of *CASP8* knockdown was greater than that of *FAT1* (e.g. SJG-8, SJG-32, which harbour *FAT1* mutations), whereas in others knockdown of *CASP8* and *FAT1* individually or in combination was equally effective (SJG-6, SJG-17).

**Fig. 4:**
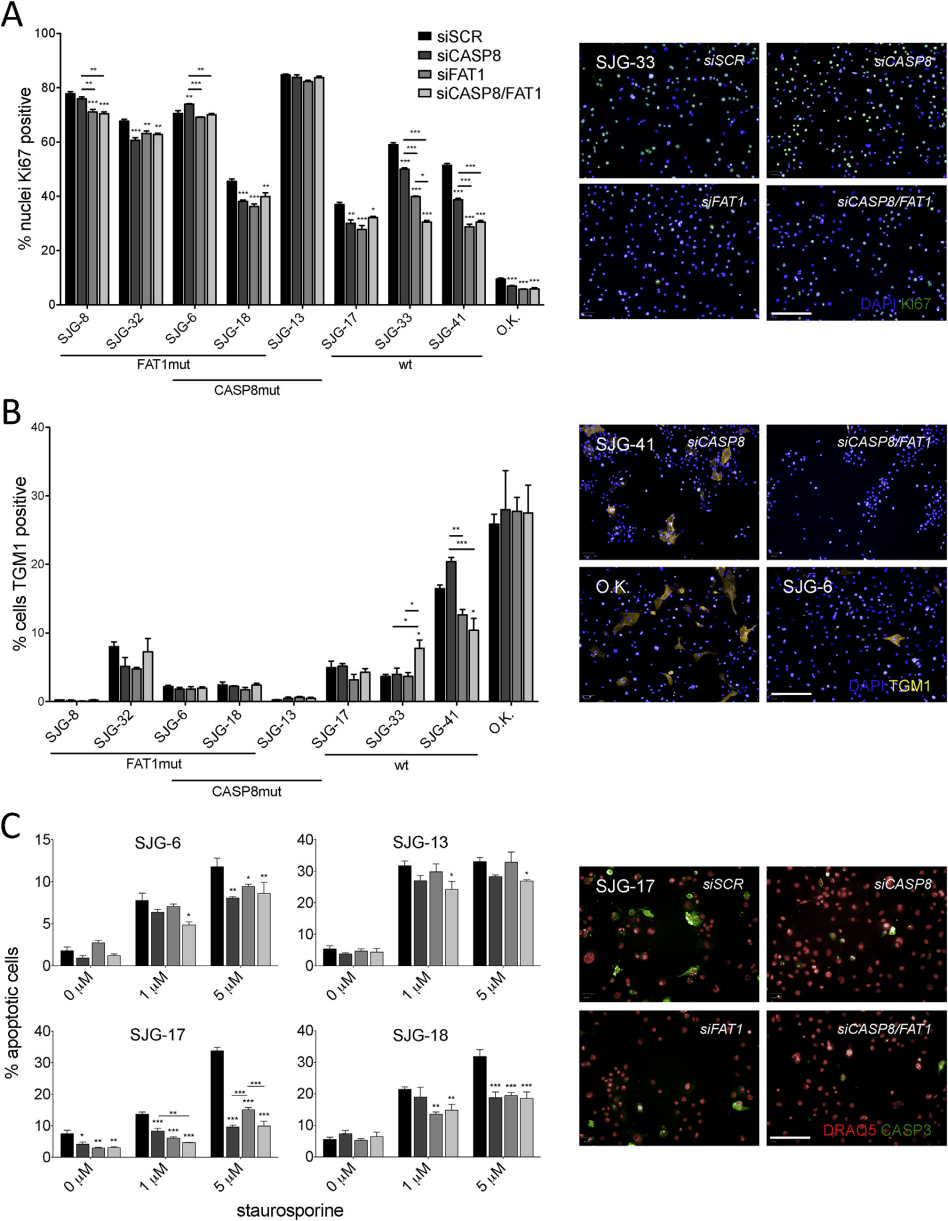
Impact of *CASP8* and *FAT1* siRNA knockdown on proliferation, differentiation and apoptosis. A) Proliferation was assessed by Ki67 immunostaining. Significant differences from non-targeting control siRNA (siSCR) are shown. wt = wild type, mut = mutant, OK = primary oral keratinocytes. B) Terminal differentiation was assessed by transglutaminase 1 (TGM1) immunostaining. A, B) Plasma membranes and nuclei were counterstained with CellMask™ Deep Red and DAPI. C) Cells were incubated with Staurosporine, and apoptosis was measured by Caspase-3 (CASP3) immunostaining. (A–C) Representative images are shown for SJG-33 (A), control O.K. and SJG-6 (B), siCASP8 and siCASP8/FAT1 SJG-41 (B) and SJG-17 (C). Data are presented as mean ± SEM. *P* value was determined by ANOVA and Tukey's test. *P < 0.01–0.05, **P < 0.001–0.01, ***P < 0.0001–0.001. Scale bars 100 μm.

The impact of *CASP8* and *FAT1* knockdown on clonal growth could not be accounted for by an increase in proliferation, and in several cases Ki67 labelling was, rather, reduced (Fig. 4A). Knockdown of *CASP8* and *FAT1* did not have a consistent effect on the proportion of cells that underwent terminal differentiation, as judged by the percentage of cells expressing Transglutaminase 1: there was no effect of double knockdown on SJG-17 cells; differentiation was stimulated in SJG-33 cells; and decreased in SJG-41 cells (Fig. 4B). Knockdown of *CASP8* and *FAT1* did, however, increase resistance to Staurosporine-induced apoptosis (Supplementary Fig. 4), as judged by Caspase-3 immunostaining (Fig. 4C).

We conclude that loss of function mutations in *CASP8* and *FAT1* are likely to confer a growth advantage on OSCC by reducing cell–cell adhesion, promoting cell migration and conferring resistance to apoptosis. Furthermore, as expected, knockdown tended to have more pronounced effects in cells with wild type *CASP8* and *FAT1*.

## Discussion

While many investigators have generated cell lines from human HNSCC and used them to study the biology of this tumour type [14], [15], [16], our study is the first to derive cell lines for which matching normal patient DNA is available. Furthermore, we demonstrate that the spectrum of tumour-acquired mutations is representative of that found in tumour biopsies, for example in the case of *NOTCH1-3* [6], [15]. These lines will be of considerable value for studying tumour cell behaviour *in vitro* and in xenograft assays.

As proof of the utility of the lines, we studied the impact of loss of functional *CASP8* and *FAT1*, two genetic changes that, we found, frequently occur together in individual OSCC. We found that in the lines, as in primary tumours, inactivating mutations in *CASP8* did not result in a reduction in *CASP8* expression, whereas *FAT1* mutation did correlate with reduced mRNA levels. While *FAT1* mutant lines showed reduced intercellular adhesion, *FAT1* knockdown in a wild type line was not sufficient to cause a significant change in morphology. It will therefore be of interest to discover whether the effect of *FAT1* loss is dependent on other OSCC mutations (Fig. 1D). For example, mutations in *PTEN* will affect the actin cytoskeleton [31] and *AJUBA* mutations are likely to impact E-cadherin mediated cell adhesion [32]. Furthermore, although we have not explored the underlying mechanism by which *FAT1* mRNA levels increase in some cell lines with increasing cell density but not in others (Fig. 3B), this has the potential to affect cell behaviour via an impact on HIPPO signalling [30].

Knockdown of each gene in cells that were wild type for *CASP8* and *FAT1* confirmed a clonal growth advantage, consistent with the observation that *CASP8* mutant cells are more aggressive in tongue xenografts [15]. This could be attributed, at least in part, to increased cell migration and reduced apoptosis, rather than to an increase in the proportion of actively cycling cells. The consequences of stimulation of terminal differentiation, observed in some cells, remain to be explored but could potentially lead to increased release of cytokines such as IL-6 that render the microenvironment permissive for tumour growth [33] and altered inflammatory signalling in *CASP8* mutant cells [19]. Cells that show a more pronounced phenotype on combined knockdown of *CASP8* and *FAT1* will be useful for exploring the cancer relevance of the observed antagonistic interactions between FAT1 and Caspase 8 [20].

In conclusion, OSCC is highly heterogeneous at both the cellular and genetic levels. The panel of cell lines that we have characterised will be useful for identifying the signalling pathways that are affected by particular combinations of mutations and for discovering how they act both at the level of the cells that bear the mutations and at the level of the surrounding stroma and immune infiltrate. In the long term, this type of approach has the potential to suggest new combination therapies to improve the outcome for patients with OSCC.

## Appendix A Supplementary data

**Supplementary Figure S1**: Whole exome sequencing coverage and depth. A) Distribution of coverage. Box-and-whisker plots display the average percentage of targeted bases at various fold-coverages across 16 tumor-derived cell lines (T) and normal patient-matched blood (N). B) Distribution of depth. Box-and-whisker plots display the average sequencing depth across all targeted regions.

**Supplementary Figure S2**: Sanger sequencing of OSCC lines identifies novel mutations in *NOTCH1-3*. Numbers represent tissue ID of cell lines, including those for which patient-matched blood was not available. Triangles represent localization of mutation in protein. N^ECD^ = Notch extracellular domain, N^TMIC^ = Notch transmembrane and intracellular domain, EGF = epidermal growth factor-like repeats, NRR = negative regulatory region, LNR = Lin-12/Notch repeats, HD = heterodimerization domain, TMD = transmembrane domain, RAM = Rbp-associated molecule domain, ANK = ankyrin repeat domain, PEST = proline, glutamic acid, serine and threonine sequence.

**Supplementary Figure S3**: A, B) siRNA knockdown efficiency. Quantitative real-time RT-PCR was performed on transcripts harvested 48 hours after transfection. LF = Lipofectamine. (B) SJG-33 cells. C) Representative colony formation assays.

**Supplementary Figure S4**: Staurosporine-induced apoptosis assay. A) Representative images of SJG-6 cells treated with varying concentrations of Staurosporine for 4 hours in complete FAD medium and stained for active Caspase-3. B-C) Dose response curves for Staurosporine treatment (B) using positive Caspase-3 as a readout for apoptotic cells and (C) measuring nuclear fragmentation index and mean nucleus area with the “Apoptosis-1” module on Harmony software. Scale bars 100 μm.

**Supplementary Table S1**: Clinical and genomic information for OSCC patient-derived cell lines. Alcohol and smoking histories were self-reported. All other information was reported by the patient’s providers. Statistics listed are for somatic mutations. Alcohol: No = does not drink, Minimal ≤ 1 drink per day for women or 2 drinks for men, Moderate = 5-7 drinks per week for women or 10-14 drinks for men, Heavy ≥ 8 drinks per week for women or 15 for men. Anatomical site: RMT = retromolar trigone. Differentiation: describes tumor biopsy from which lines were derived. Mb = megabase.

**Supplementary Table S2**: Somatic mutations in OSCC lines. Mutations presented were detected by whole exome sequencing and annotated using ANNOVAR.

**Supplementary Tables S3-4**: Significantly mutated pathways in OSCC lines. Mutated KEGG pathways were identified using (S3) the Oncodrive-fm method [21] or (S4) via pathway enrichment analysis.
