# Supplementary material for: Integrative genomic and functional analysis of human oral squamous cell carcinoma cell lines reveals synergistic effects of FAT1 and CASP8 inactivation
Source: Cancer Lett. 2016 Dec 1;383(1):106–14. doi: 10.1016/j.canlet.2016.09.014 (PMC5090049; doi:10.1016/j.canlet.2016.09.014)

# Supplementary Figure S1

A

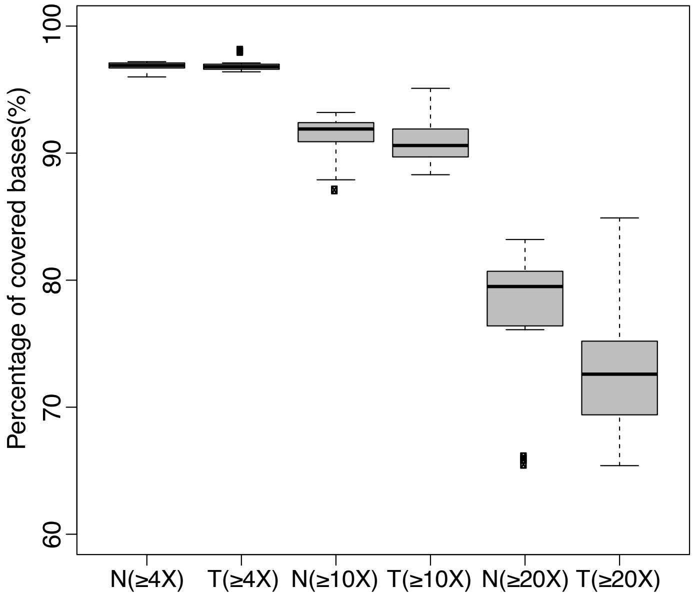


B

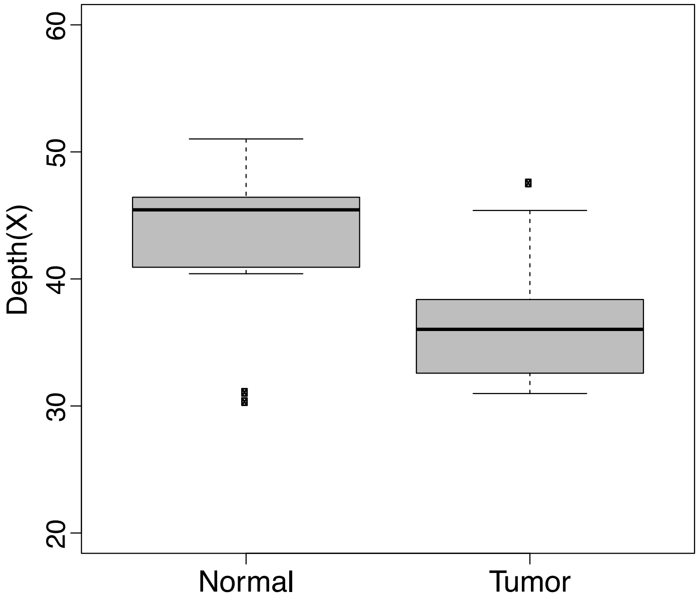

# Supplementary Figure S2

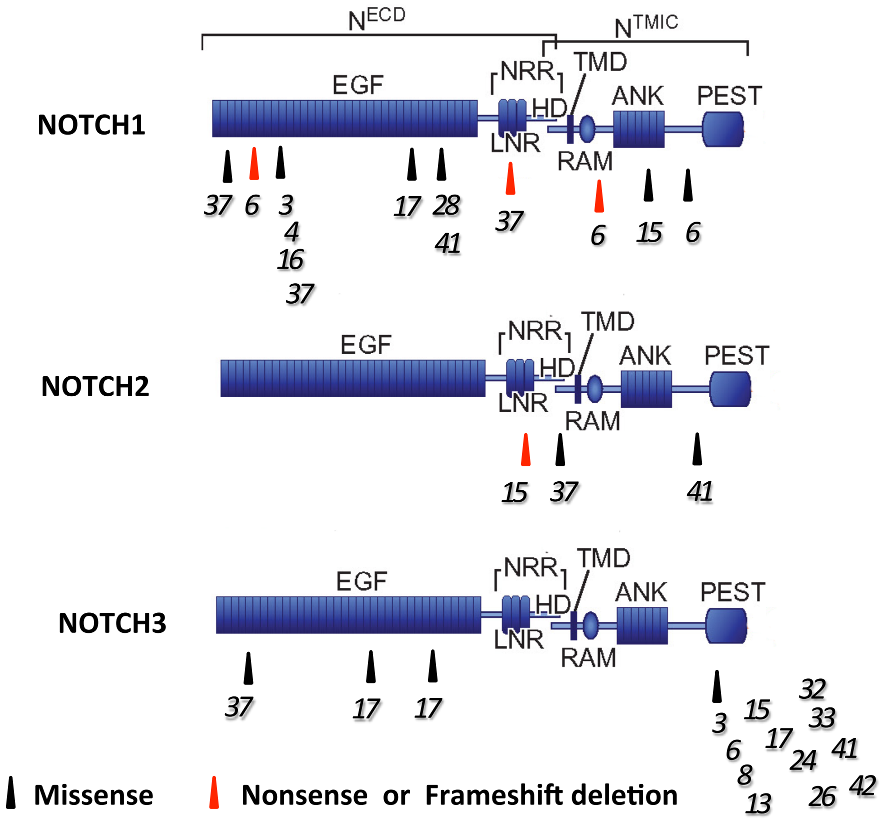

# Supplementary Figure S3

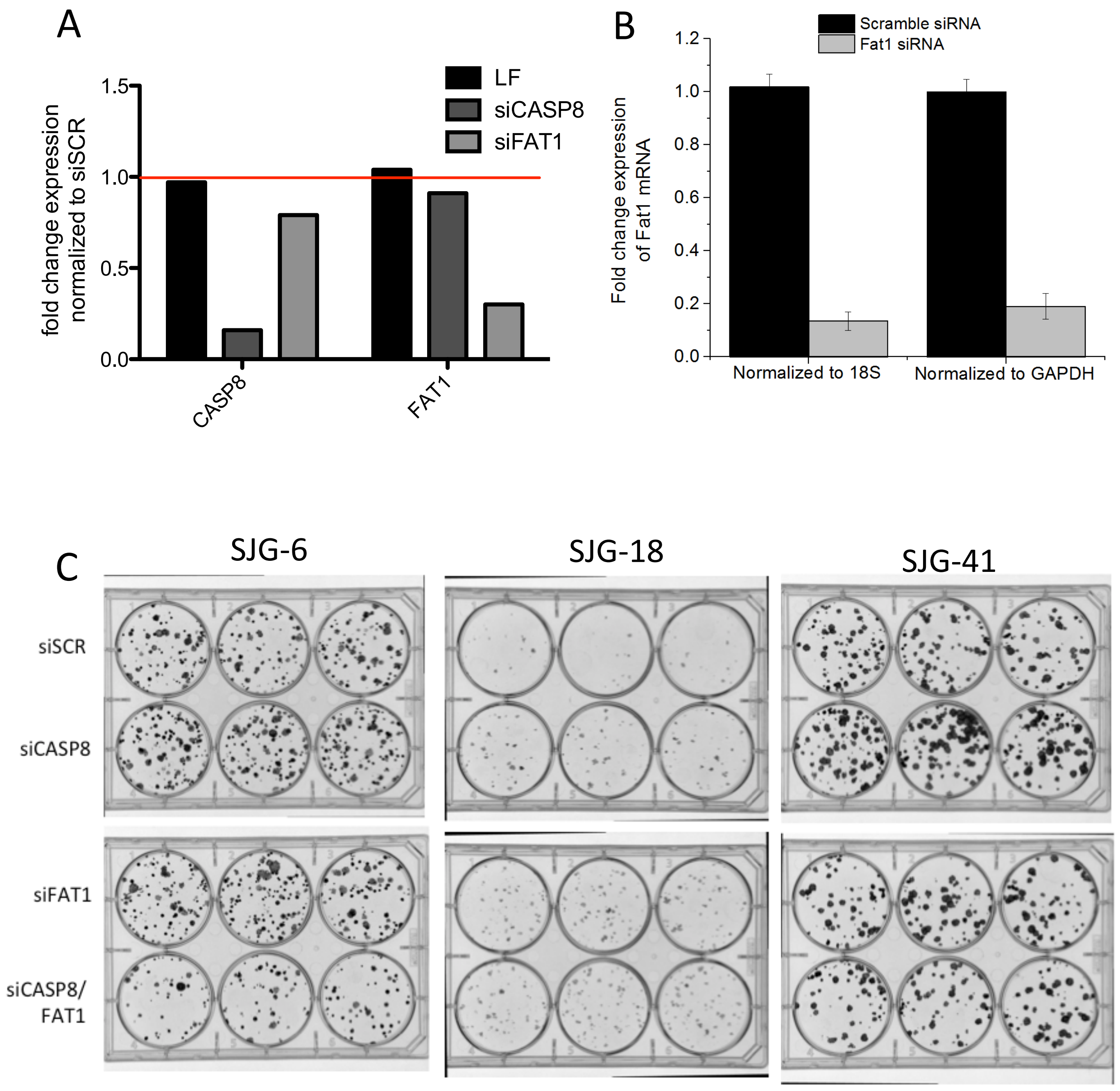

# Supplementary Figure S4

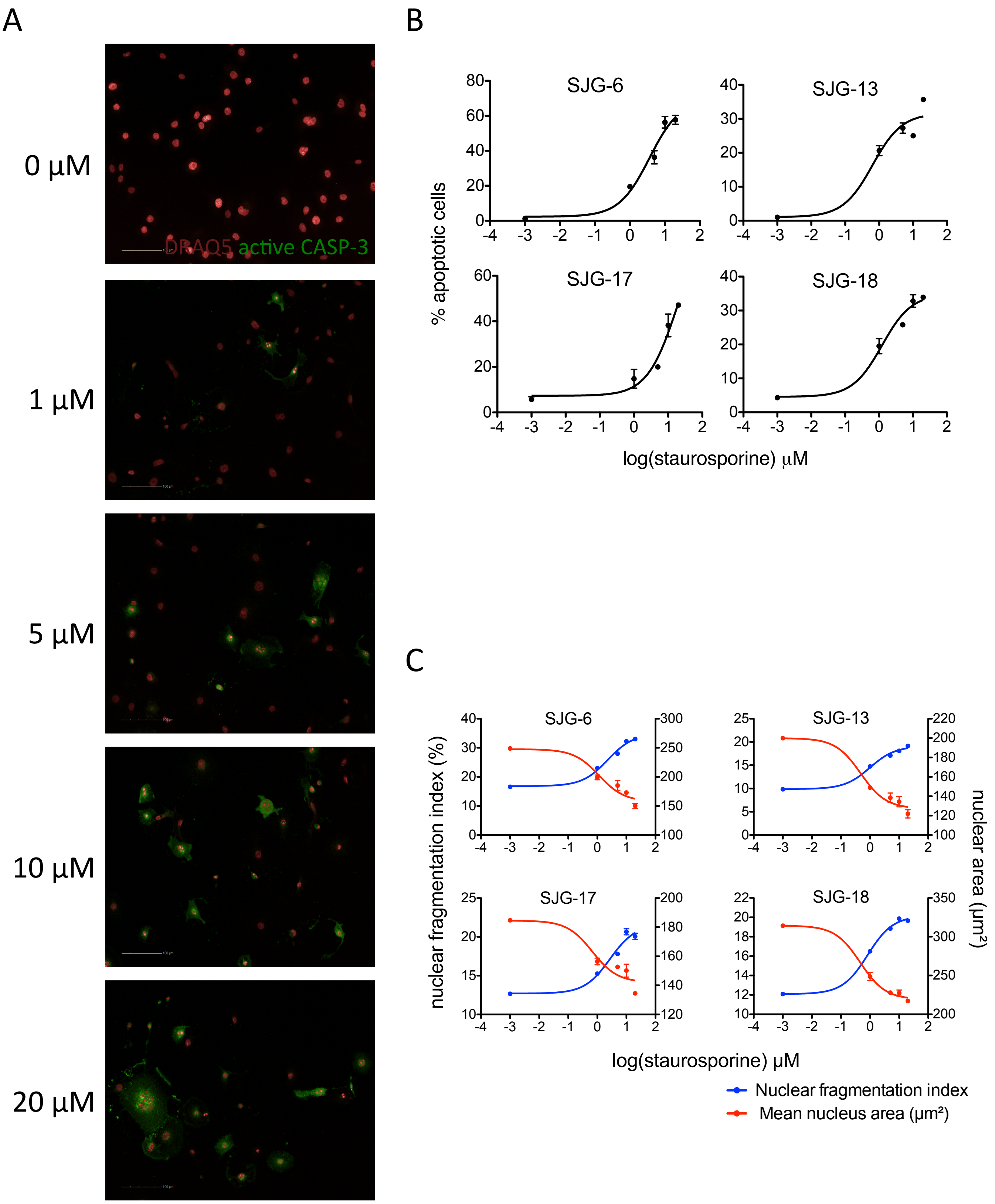

Supplement: Supplementary file 1 [file mmc1.pdf]
